# In Vivo Biological Evaluation of Biodegradable Nanofibrous Membranes Incorporated with Antibiofilm Compounds

**DOI:** 10.3390/polym13152457

**Published:** 2021-07-26

**Authors:** Thaise C. Geremias, Suelen C. Sartoretto, Marcos A. Batistella, Antônio A. Ulson de Souza, Adriana T. N. N. Alves, Marcelo J.P. Uzeda, Jose Calasans-Maia, Pietro Montemezzi, Carlos Fernando de Almeida Barros Mourão, Monica Calasans-Maia

**Affiliations:** 1 Clinical Research Laboratory in Dentistry, Universidade Federal Fluminense, Niteroi 24020-140, Brazil; thaisecg@id.uff.br (T.C.G.); susartoretto@hotmail.com (S.C.S.); aterezinhanovellino@gmail.com (A.T.N.N.A.); 2 Oral Surgery Department, Dentistry School, Universidade Veiga de Almeida, Rio de Janeiro 20271-020, Brazil; 3 Laboratory of Mass Transfer, Department Chemical Engineering and Food Engineering, Universidade Federal de Santa Catarina, Florianópolis 88040-970, Brazil; marcos.batistella@mines-ales.fr (M.A.B.); antonio.augusto.souza@ufsc.br (A.A.U.d.S.); 4 Polymers Composites and Hybrids (PCH), IMT Mines Ales, 6 Avenue des Clavières, 30100 Alès, France; 5 Oral Diagnosis Department, Universidade Federal Fluminense, Niteroi 24020-140, Brazil; 6 Oral Surgery Department, Dentistry School, Universidade Iguaçu, Nova Iguaçu 26260-046, Brazil; mjuzeda@oi.com; 7 Oral Surgery Department, Dentistry School, Universidade Federal Fluminense, Niteroi 24020-140, Brazil; 8 Orthodontics Department, Universidade Federal Fluminense, Niteroi 24020-140, Brazil; josecalasans@id.uff.br; 9 Independent Researcher, 24128 Bergamo, Italy; m.montemezzi@libero.it; 10 Graduate Program in Biotechnology, Universidade Federal Fluminense, Niteroi 24020-140, Brazil

**Keywords:** antibiofilm activity, polymers, bacterial adhesion, quorum sensing, PLGA, electrospinning

## Abstract

Guided bone regeneration involves excluding non-osteogenic cells from the surrounding soft tissues and allowing osteogenic cells originating from native bone to inhabit the defect. The aim of this work was to fabricate, analyze antibiofilm activity and evaluate in vivo biological response of poly (lactic-*co*-glycolic acid) (PLGA) electrospun membranes incorporated with tea tree oil and furan-2(5*H*)-one. Samples were exposed to *Streptococcus* *mutans* culture and after 48 h incubation, biofilm was evaluated by colony forming units (CFU/mL) followed by scanning electron microscopy. Additionally, seventy-five Balb-C mice were divided into five experimental groups for subcutaneous implantation: tea tree oil loaded PLGA electrospun fiber membrane, furanone loaded PLGA electrospun fiber membrane, neat PLGA electrospun fiber membrane, a commercially available PLGA membrane –Pratix^®^ and Sham (no-membrane implantation). Post implantation period of each experimental group (1, 3 and 9 weeks), samples were collected and processed for by histological descriptive and semiquantitative evaluation. Results showed a significant reduction of bacterial attachment on tea tree oil and furan-2(5*H)-*one incorporated membranes. Macrophage counts were significant found in all the materials implanted, although giant cells were predominantly associated with electrospun fiber membranes. The incorporation of antibiofilm compounds in nanofibers membranes did not incite inflammatory response significantly different in comparison with pure PLGA electrospun membranes, indicating its potential for development of novel functionalized membranes targeting the inhibition of bacterial biofilms on membrane-grafting materials.

## 1. Introduction

Augmentations of alveolar ridge defects, implant wound healing and regeneration of periodontal tissue defects may represent few clinical scenarios of bone tissue insufficiency in which guided bone regeneration (GBR) technique has proved to be a very suitable treatment. In such bone repair method, membrane-grafting materials association can establish a protected environment for blood clot and competent regenerative cells by the interposition of a physical barrier between the soft tissues of the flap and the osseous defect and allow the angiogenesis and osteogenesis [1,2,3,4,5].

In order to prevent epithelial and connective tissue cells from invading the bone tissue regenerative area, membranes may differ in composition, from natural to synthetic materials, and present different structures. Considering that nonresorbable membranes have exhibited a range of complications up to 45.5%—including dehiscence and premature exposition [6], added to a necessity for a removal surgery, implicating in additional pain, discomfort and in an economic burden [7], absorbable materials have been elected as an alternative approach. The most commonly used biodegradable synthetic polymers for tissue engineering are saturated poly(α-hydroxy esters), including poly(lactic-*co*-glycolic) (PLGA) copolymers, which provides superior control regarding degradation properties by varying the ratio between its monomers [8] and several functionalize possibilities.

Barrier membranes considered ideal should act also as substrate for tissue attachment alongside with promotion of the progenitor cells [9]. In an effort to mimic extra cellular matrix (ECM) native protein structure and provide high surface area to volume ratio, numerous research have explored the electrospinning process for biomaterial engineering [10,11,12,13]. Indeed, electrospun fibers stimulate positive cell–ECM interactions, support cell attachment, enhance proliferation rate, maintain cell phenotype, and activate cell-signaling pathways by providing physical and chemical stimuli to cells [14].

Despite the clinical success of the GBR technique, one of its main causes of failure is related to early or late exposure of barrier membrane, leading to contamination and infection of the biomaterial, compromising bone regeneration [15,16]. Bone infections represent a complicated treatment due to multiple biological factors, and its vascular undersupply makes it much more difficult for systemic therapies and host immune cells to reach and envelop the infected area with efficient metabolic response to infection [17]. In addition, following bacterial attachment, bacteria begin to produce a protective layer (extracellular polymeric substance) that allows for immune evasions as well as resistance of antimicrobial agents [18,19,20].

The rising prevalence of infectious diseases caused by multidrug-resistant microorganisms [21] is leading to a major research effort to find alternative antimicrobial approaches, natural-based compounds [22,23] or antibiotic-free therapeutics that target pathogenicity control bacterial adherence to surfaces as well as signaling systems controlling bacterial group behavior organized in biofilms [24,25,26].

Several compounds have been recently tested for antibiofilm effects, acting through bacterial-communication system (so called quorum-sensing), which orchestrate collective behaviors, virulence factors and major sequence events associated with infection [27,28,29]. Furanone, isolated from marine red algae *Delisea Fulchra*, and its synthetic derivatives appear to be the most effective on preventing bacterial adherence and biofilm formation, showing results on *Klebsiella pneumoniae* [30], *Bacillus subtilis* [31], *P. aeruginosa* [32]*, F. nucleatum, Porphyromonas gingivalis*, *Tannerella forsythia* [33] and *Streptococcus mutans* [34]. Studies have reported similar activities for *melaleuca alternifolia* extract—tea tree oil, that exhibited biofilm inhibition on *S. mutans* [35], *P aeruginosa* [36] and oral polymicrobial biofilm in situ [37].

These findings have prompted our group into produce functionalized resorbable membranes incorporating antibiofilm that minimize initial bacterial adhesion, preventing further infections, while promoting integration with host tissues, since biologic behavior of barrier membranes is a supreme element for the primary evaluation of regenerative materials [5]_._ Considering that *Streptococci* and *Actinomyces* represent major initial colonizer of oral surface [38], *S*. *mutans* biofilm experimental model has been selected on that basis.

The purpose of this present study was to evaluate the biocompatibility of a novel biodegradable nanofibrous PLGA membranes incorporated with antibiofilm, furanone and tea tree, on its most effective concentration, in subcutaneous rat model, according to our previous study [39]. To the best of our knowledge, this is the first manuscript on incorporating these compounds in fibrous structures and investigating its antibiofilm activity and in vivo biological response for biomedical applications.

## 2. Materials and Methods

### 2.1. Electrospinning Materials

Medical grade PLGA copolymer (82:18 molar ratio, inherent viscosity midpoint of 1.8 dlg^−1^) was purchased from Corbion Purac (PURASORB^®^ PLG 8218, Amsterdam, Netherlands). Australian *Melaleuca alternifolia* (tea tree) essential oil (Desert Essence, Hauppauge, NY, USA) and furan-2(5*H*)-one were purchased from Sigma-Aldrich (St. Louis, MO, USA). Chloroform (Vetec, Duque de Caxians, Brazil) and *N,N*-dimethylformamide (DMF) (LabSynth, Diadema, Brazil) were used as solvent. All solvents were of analytical grade.

### 2.2. Electrospinning Process

PLGA was dissolved (10 *w*/*v*%) in a solution containing chloroform and *N,N-*dimethylformamide (95:5 *v/v*) for 8 h under constant stirring at room temperature to produce pure PLGA electrospun membranes. For the tea tree essential oil and furanone loaded PLGA solutions, each agent to be tested was added separately into the polymer solution at 0.004% (*v/v*), according to previous study [39]. PLGA membranes, with no compound’s incorporation were also prepared. The prepared solutions were then stirred at room temperature to obtain homogeneous solution. The electrospinning process was carried out under stable conditions (temperature 25 °C and relative humidity 65%). Each electrospinning solution was loaded separately into a 5.0 mL plastic syringe equipped with a 23 G stainless steel needle, which was connected to a high voltage power supply, applying a voltage of +13 kV. The solutions were fed at 2 mL h^−1^. An aluminum sheet wrapped around the rectangular collector was placed at 15 cm from the needle tip to the collector. Electrospinning process was carried out for 3 min, while the collector was rotating at 500 rpm. All samples were stored at 5 °C in lightning protection conditions for further analyses.

### 2.3. Other Biomaterials

A commercially available poly (lactic-*co*-glycolic acid) dental membrane (Pratix^®^, Baumer, Mogi Mirim, Brazil) was purchased for comparison in the subcutis tissue of rats.

### 2.4. Bacterial Strains and Growth Condition

Strains of *S*. *mutans* (ATCC 25175) were grown under microaerophilic conditions for 48 h at 37 °C on Brain Heart Infusion (BHI) agar plates (Difco, Franklin Lakes, NJ, USA), supplemented with 3 g/L of yeast extract (Difco, Franklin Lakes, NJ, USA) and 200 g/L of sucrose (Difco, Franklin Lakes, NJ, USA). For biofilm assays, *S. mutans* cells were inoculated in Tryptic Soy Broth (TSB) (Difco, Franklin Lakes, NJ, USA), supplemented with 3 g/L of yeast extract and 200 g/L of sucrose, and incubated for 18 h at 37 °C. The cells were, then, harvested by centrifugation at 5000 rpm for 10 min at 4 °C, washed twice in a phosphate buffer solution (PBS) and re-suspended in TBS medium supplemented with mucin (2,5 g/L), peptone (5 g/L), urea (1 g/L), yeast extract (2 g/L) and sucrose (200 g/L).

### 2.5. Biofilm Formation and Assay

To assess the antibiofilm activity of electrospun fiber membranes, the samples were cut to their proper size to cover the 24-well plates and sterilized by UV irradiation for 30 min preceding the experiment. Then, the sterile samples were placed into 24-well plates containing 2 mL of *S*. *mutans* suspension in TSB medium and incubated for 48 h at 37 °C, under microaerophilic conditions.

Biofilm formation was quantified on electrospun membranes after incubation with *S. mutans*. These membranes were washed twice with PBS and adherent bacteria were detached from the membrane surfaces by 1% proteinase K treatment for 60 min (Sigma-Aldrich, St. Louis, MO, USA). Subsequently, a physical method was also used to increase biofilm detachment. Adherent bacteria were removed by vortex treatment for 1 min. The suspensions were then serially diluted (up to 10^−6^) in PBS and plated on BHI agar (100 μL) and incubated as previously described, to quantify the CFUs/mL.

These experiments were performed in triplicate and carried out in three independent assays.

### 2.6. Surface Analysis of Electrospun Membranes

The synthesized electrospun membranes were prepared and were sputter-coated with a thin layer of gold/palladium prior to Scanning Electron Microscopy (SEM) analysis (JEOL JSM-6390LV, Japan) at 15 kV.

For microscopic analyses of surfaces covered with biofilms, the membranes were washed twice in PBS and fixed in glutaraldehyde 2% for 5 min. Then, surfaces were washed three times in PBS and dehydrated through a series of graded ethanol solutions (50%, 70%, 80%, 90%, and 100%). Samples covered with *S. mutans* biofilms were then sputter-coated with gold and analyzed by Scanning Electron Microscopy.

### 2.7. Surface Morphology and Fibers Diameter

Diameter distribution, average diameter, and pore size of electrospun fibers were estimated from SEM micrographs via ImageJ analysis software (NIH, Bethesda, MD, USA). Averages were obtained from the measures randomly at 50 different points for each sample.

### 2.8. Experimental Groups

For subcutis implantation, the membranes evaluated were divided into: Group 1—Electrospun PLGA loaded with tea tree essential oil; Group 2—Electrospun PLGA loaded with furanone; Group 3—Electrospun pure PLGA and Group 4—Pratix^®^ membrane. Was also included a Sham group (no-membrane implantation)—Group 5.

### 2.9. Ethical Principles and Conduct in the Care and Use of Animals

This study was carried out in compliance with the guidelines of the 3Rs Program (Replacement, Refinement and Reduction), whose objective is to reduce the number of animals used during experimentation, to minimize pain and discomfort (National Center for the Replacement, Refinement & Reduction:NC3Rs, 2010). The experiments were reported according to the ARRIVE (Animal Research: Reporting of In Vivo Experiments) [40] and Planning Research and PREPARE (Experimental Procedures on Animals: Recommendation for Excellence) guidelines [41] regarding relevant items. Furthermore, this study was approved by The Ethical Committee of the Universidade Federal Fluminense, under the protocol CEUA/UFF: 980. The animal breeding and experiments were performed according to conventional guidelines of the NIH (National Institutes of Health) Guide for the Care and Use of Laboratory Animals following the Brazilian Directive for the Care and Use of Animals for Scientific and Didactic Purposes—DBCA and the CONCEA (Conselho Nacional de Controle e Experimentação animal) Euthanasia Practice Guidelines.

### 2.10. Animals Welfare

During the experimental period, the animals were housed at the Laboratory of Animal Experimentation (LEA), at the Universidade Federal Fluminense (UFF), in special isolators created for this animal’s research. Each of the isolator housed a maximum of five mice and were prepared with dry wood—pine, shavings. A standard diet consisting of ground rations (Nuvilab^®^, Curitiba, Brazil), changed daily to mitigate fungi and bacteria contamination, was administered, alongside with water through glass beakers with stainless steel spouts. Special arrangements were established to maintain ideal room temperature, around 20 °C and 22 °C, to ensure animals health, comfort, and welfare. Such conditions also imply on the correct metabolic cycle for these experiments, and guarantee animals’ growth and control of the photoperiod, 12–12 h light/dark. Moreover, a senior veterinarian supervised proper animal care throughout pre and postoperative period.

### 2.11. Characterization of Animal

Seventy-five Balb-C mice, male and female, weighing approximately 30 g, were provided by the Laboratory Animal Center (NAL) of the Universidade Federal Fluminense (NAL-UFF) for this study. The animals were randomly divided, through a random draw (using an opaque envelope containing the group name) into five groups: G1, G2, G3, G4 and G5, Sham group (control). The groups were, then, subdivided according to experimental periods of evaluation (1, 3 and 9 weeks), with five animals in each group/experimental period. The level of significance of 5% and a power test of 80% was used to calculate the sample size used in this study [Sealedenvelope. Available online: https://www.stat.ubc.ca/~rollin/stats/ssize/n2.html, accessed on 2 March 2019 [42], which suggested five animals in each group.

### 2.12. Anesthesia and Surgical Procedures

Followed by a period of 24-h fasting, all animals were submitted to general intraperitoneal anesthesia, in accordance with Fluminense Federal University protocol. A 21-G needle (Becton-Dickinson^®^,Juiz de Fora, Brazil) was prepared with a solution of 1.0 mL of 10% ketamine hydrochloride (Dopalen^®^−100 mg/mL Ceva, Paulina, Brazil), 0.5 mL of 2% xylazine (Anasedan^®^−20 mg/mL Ceva, Paulina, Brazil), and 8.5 mL of sterile saline solution (KabiPac^®^, Fresenius Kabi Brasil Ltd., Barueri, Brazil), and 0.2 mL of the anesthetic solution in the animal’s lower left quadrant was injected.

**Figure 1:**
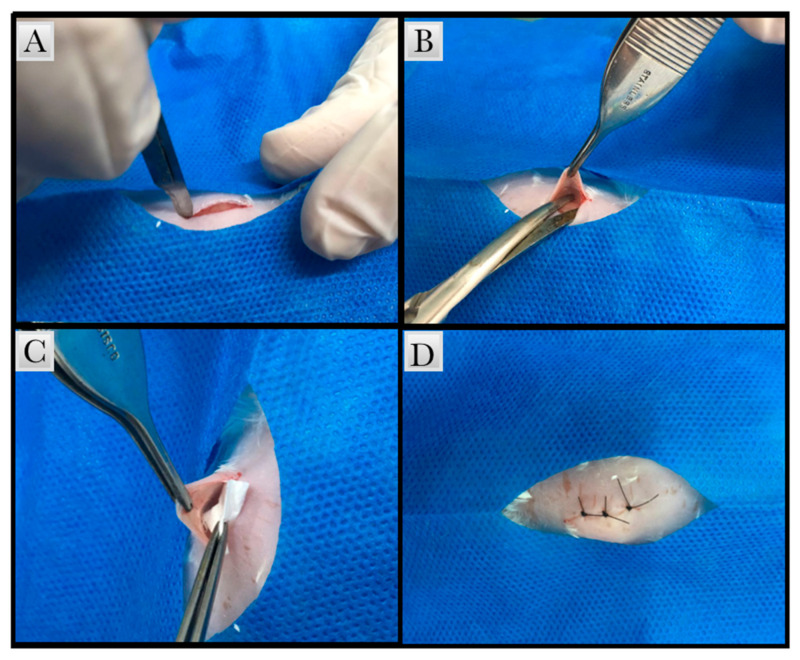
Surgical procedure (**A**) Subcutaneous linear incisions were made using a no. 15 C scalpel blade in the dorsal region of the animal. (**B**) Divulsion of the subcutaneous tissue was performed with a blunt-tipped scissors. (**C**) Membrane insertion (**D**) Suture of the implantation area with 5-0 Nylon.

Immediately after pain reflex absence, trichotomy was performed using sterile razor blades, then a chlorhexidine degermant solution was applied subsequently to a 2% alcoholic chlorhexidine (Rioquímica, São José do Rio Preto, Brasil) solution. Subcutaneous linear incisions were made on animal’s dorsal region using a no. 15 C scalpel blade (Becton-Dickinson^®^, Juiz de Fora, Brazil), and divulsion of the subcutaneous tissue was performed with a blunt-tipped scissors (Metzenbaum 15 cm Colgran, São Caetano do Sul, Brazil) to insert the testing membranes material (1 cm), as shown in Figure 1. The incisions were then closed using a 5-0 Nylon suture (Ethicon^®^, Johnson & Johnson, São Paulo, Brazil), followed by antisepsis with the 2% alcoholic chlorhexidine solution.

In concern to Sham experimental group, the same procedures (incision, displacement, and suturing) were performed, although, no membrane was implanted

After surgery, animals were housed in isolators (5 animals each), separated according to their experimental group, and were strictly monitored during anesthesia recovery, to avoid injuries due to compromised coordination. Meloxicam 5 mg/kg (Eurofarma, Itapevi, Brazil) therapy was given post-surgery procedure and for the following 2 days, once daily. Postoperative care and monitoring for any relevant complication were carried out.

### 2.13. Samples Collection and Materials Processing

After experimental periods of 1, 3 and 9 weeks, the mice were euthanized by a lethal dose of 150 mg/kg pentobarbital and lidocaine anesthetic solution, in accordance with the recommendations of National Resolution No. 13 of the National Council for the Control of Animal Experimentation (CONCEA). An excisional biopsy of the implant area and surrounding tissue was performed with safety margin of 5 mm, using a no. 15 C scalpel blade (Becton-Dickson, Juiz de Fora, Brazil), and a pair of blunt-tipped scissors (Golgran, São Caetano do Sul, Brazil). Sham group also received a lethal anesthesia dose, and the biopsy were performed at incision sites. All the obtained samples were fixed in 4% formaldehyde (phosphate buffer, pH 7.4) for at least 24 h.

Following fixation period, samples were then dehydrated in ascending series of 80%, 95% and 100% ethanol baths, and finally immersed in liquid paraffin and embedded into blocks. The blocks were cut using a standard microtome (Leica RM 2250) perpendicular to the plane of the skin for the subcutis implants into 5 μm thick histology sections. The sections were mounted on microscopy glass slides and stained with Hematoxylin/Eosin (HE).

### 2.14. Microscopic Descriptive Analysis

The stained samples were scanned and observed using a light field microscope (OLYMPUS BX43^®^, Tokyo, Japan) for descriptive histological analysis. Selected images were captured thorough a microscope-coupled camera (OLYMPUS SC100^®^, Tokyo, Japan) associated with a high-resolution software (CellSens^®^1.9 Digital Image, Tokyo, Japan). A ×40 magnification was used for comprehensive scanning of the area of interest and ×200/400 magnification were applied to obtain improved cellular and tissue details. An Inflammatory portrayal at membrane–tissue interface, including connective tissue, inflammatory infiltrate, and fibrosis, as well as implanted membrane degradation pattern were evaluated.

### 2.15. Evaluation of the Local Biological Effects of Implantation of the Biomaterials. Semiquantitative Histological Analysis: ISO 10993-6:2016/Part 6/Annex E

All microscopic analysis was performed by a single-blinded, calibrated pathologist. The digital images of the stained slides were obtained using a light-field microscope (OLYMPUS^®^, Tokyo, Japan). A semiquantitative histological analysis was performed on each subcutaneous slide, from which 5 fields were scanned according to the area of interest, excluding any possible overlap, and captured using a high-resolution software (CELLSENS^®^1.9 Digital Image, Olympus, Tokyo, Japan), at ×400 magnification. Then, a count of the surveyed elements results in a progressive score value (ranging from 0 to 5). The biological response parameters at the tissue–membrane interface was evaluated and scored as follows:

(a) The quantity and distribution of inflammatory cells (polymorphonuclear leukocytes, lymphocytes, plasma cells, macrophages, and multi-nucleated giant cells) at the material/tissue interface.

(b) Inflammatory response parameters (neovascularization, fibrosis degree of fibrous capsule, and fatty infiltrate).

(c) The presence and extension of necrosis.

This process was performed for all the animals in every group and experimental period involved in the research. Also, an average, acquired from the sum of scores, for test groups and control group was calculated. The difference between values of the tests groups and control group (Sham) were ranked according to the following criteria: non-irritant (0.0 to 2.9), slight irritant (3.0 to 8.9), moderate irritant (9.0 to 15.0), and severe irritant (>15).

### 2.16. Statistical Analysis

Statistical analysis to evaluate the results of inflammatory cells response was performed by parametric description with means and confidence intervals (CI). The obtained data were not normal (D’Agostino-Pearson normality test), converted into a Y logarithm, and compared through ANOVA and Tukey’s post-test (*p* < 0.05).

For biofilm inhibition activity, results were analyzed by ANOVA and Tukey’s post-hoc test with a significance level of *p* < 0.01. The Prism Graph Pad 8.3 software (La Jolla Inc., Irvine, CA, USA) was used.

## 3. Results

### 3.1. Surface Morphology, Diameter Distribution and Pore Size of Electrospun Fibers

**Figure 2:**
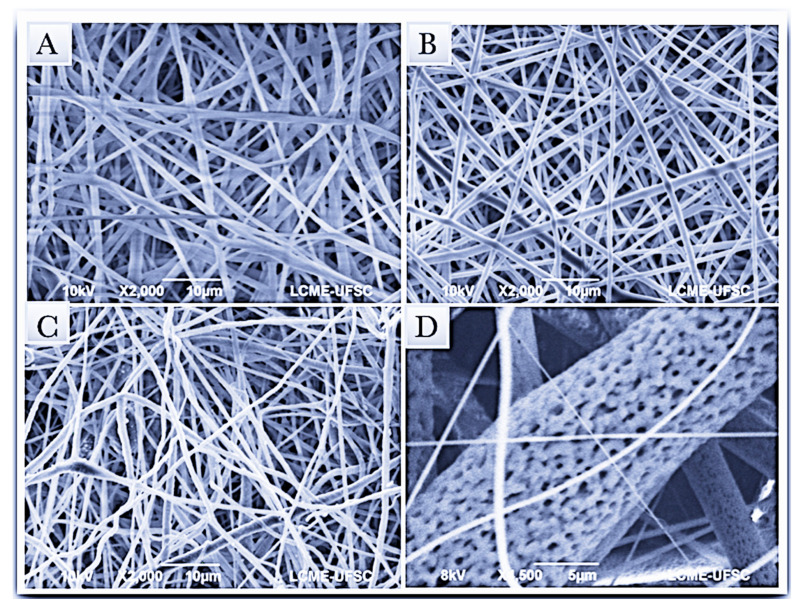
Scanning Electron Micrographs (SEM) of the electrospun membranes (**A**) Electrospun PLGA loaded with tea tree essential oil, (**B**) Electrospun PLGA loaded with furanone, (**C**) Electrospun pure PLGA. (**D**) Porous surface of the electrospun fibers.

Electrospun PLGA and PLGA fibers loaded with tea tree essential oil and furanone were fabricated by eletrospinning method. Electrospinning processing parameters were optimized at a flow rate 2 mL/h, voltage of 13 kV and distance of 15 cm and the produced membranes were analyzed by Scanning Electron Microscopy (SEM). Figure 2 exhibited SEM images that illustrates continuous, homogenous and bead-free electrospun fibers. Highly porous surface of the fibers were also revealed, obtained from electrospinning determined parameters.

**Figure 3:**
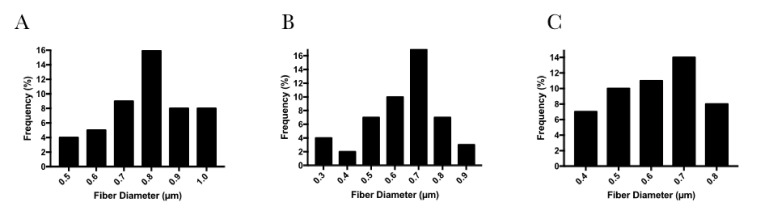
Fiber Diameter Distribution of electrospun membranes. (**A**) Electrospun PLGA loaded with tea tree essential oil, (**B**) Electrospun PLGA loaded with furanone, (**C**) Electrospun pure PLGA.

The average fiber diameters of PLGA loaded with tea tree essential oil, PLGA loaded with furanone, and pure PLGA fiber membranes were 0.78 ± 0.14, 0.63 ± 0.15 and 0.61 ± 0.13 µm, respectively. The distribution of fiber diameters (Figure 3) indicates that the addition of tea tree essential oil pointed to a slight increase in fiber diameter as compared to furanone additive and pure PLGA. Yet, neither addition did significantly affect the average of electrospun fibers.

**Figure 4:**
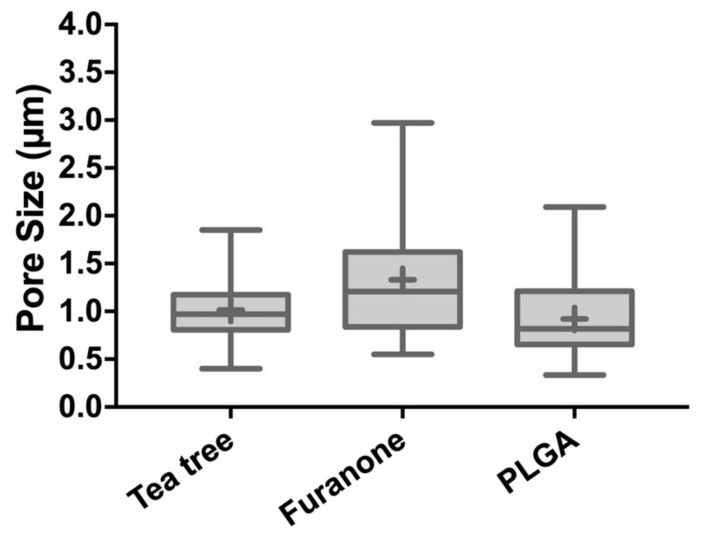
Average Pore size of PLGA loaded with tea tree essential oil, PLGA loaded with furanone, and pure PLGA fiber membranes (no statistically significant difference).

Pore sizes were analyzed and the average pore diameter of the electrospun fiber membranes are shown in Figure 4. The average pore diameters of pure PLGA electrospun fiber membranes was 0.91 ± 0.39 µm, whereas the tea tree oil addition slightly increased the pore size to 1.01 ± 0.28 µm. Furanone addition exhibited the highest measures for pore average (1.33 ± 0.59 µm) and maximum pore size of 2.97 µm. Additionally, the maximum pore size of tea tree samples (1.85 µm) and pure PLGA fiber membranes (2.09 µm) were reduced. However, no significant differences between electrospun fiber membranes were found.

### 3.2. Antibiofilm Analysis

**Figure 5:**
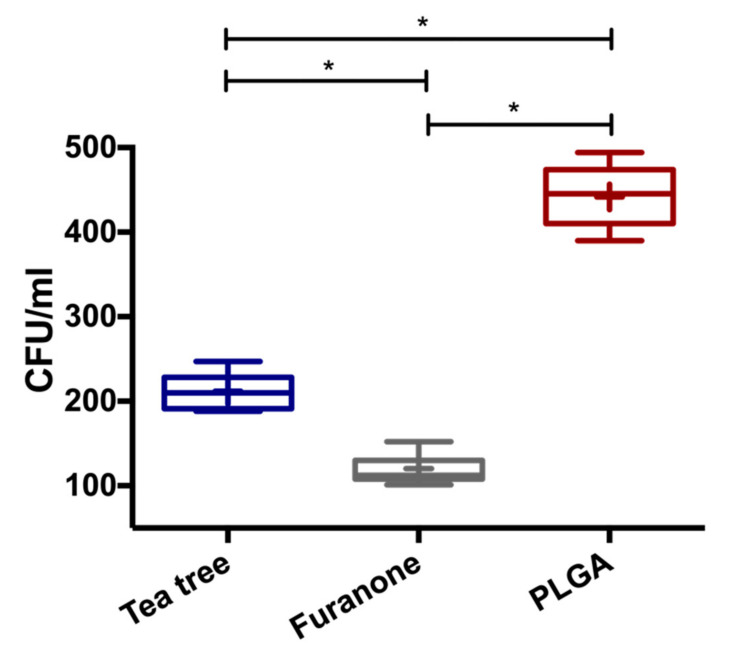
*S*. *mutans* biofilm formation (TSB, 48 h at 37 °C) over electrospun membrane surfaces, measured by colony forming units (CFU/mL). * ANOVA and Tukey’s post-hoc with a significance level of *p* < 0.01.

The antibiofilm activity was assessed by CFU/mL counting post bacterial disaggretion from the electrospun membrane surfaces. As presented in Figure 5, the mean of adhered viable cells in the *S*. *mutans* biofilm was 211 and 120 CFU/mL over tea tree and furanone PLGA loaded membranes, respectively. In contrast, pure PLGA exhibited 441 CFU/mL. During 48 h incubation, the tea tree and furanone antibiofilm membranes presented a significant decrease in bacterial attachment (*p* < 0.01).

**Figure 6:**
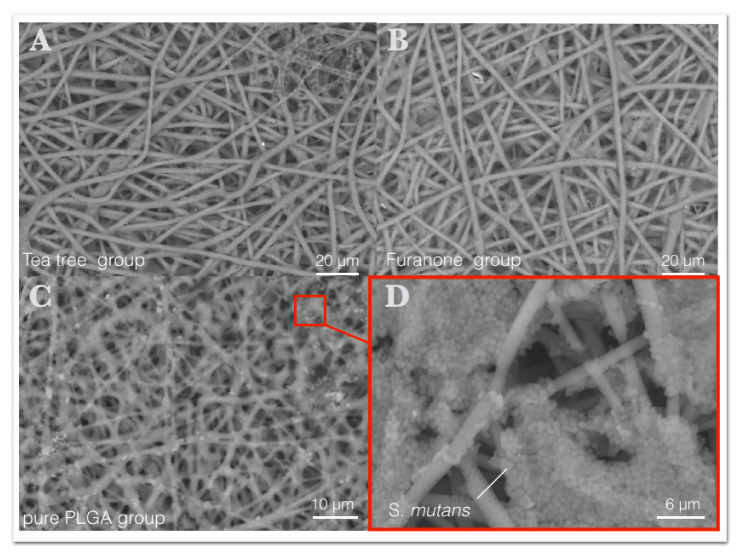
Scanning Electron Micrograph (SEM) of membrane surfaces after exposition to *S*. *mutans* cultures on biofilm formation assay (48 h in TSB, 37 °C). (**A**,**B**) Antibiofilm-incorporated electrospun fibers visibly free of *S*. *mutans* colonies. (**C**,**D**) *S*. *mutans* colonies evidenced on electrospun fiber surfaces in the absence of the biofilm inhibitors.

Scanning Electron Microscopy (SEM) images of electrospun membrane surfaces revealed the patterning of *S. mutans* biofilms formed, as shown in Figure 6A–D. A significant amount of biofilm accumulation was noticed in membrane samples corresponding to pure PLGA (Figure 6C,D) contrasting with surfaces of electrospun membranes loaded with biofilm inhibitors—tea tree and furanone, which appeared visibly free of *S. mutans* colonies (Figure 6A,B). *S. mutans* colonies had grown agglomerate on fibers surface, originated from the electrospinning process, showing itself susceptible to accumulation of *S. mutans*.

### 3.3. Microscopic Descriptive Analysis

Biological tissue response post-implantation of tea tree-based PLGA membrane (G1), furanone-based PLGA membrane (G2), pure PLGA membrane (G3), and a commercially available membrane (G4) were studied. All the animals recovered with no clinical complications and macroscopic examination presented a good wound closure without signs of infection or inflammation.

Descriptive analysis of inflammatory infiltrate and of resorption pattern was performed in 1st, 3rd and 9th week, according to the experimental periods previously determined. Sham group (G5) has not been assessed for descriptive analysis.

#### 3.3.1. 1-Week Post-Implantation

**Figure 7:**
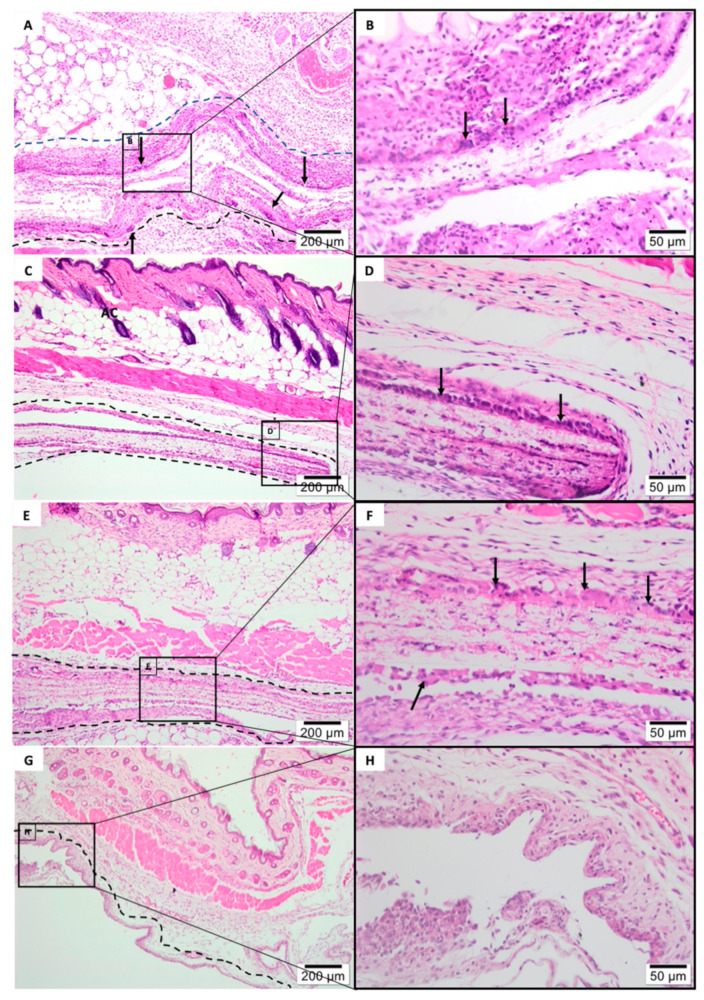
Histology of subcutaneous evaluation of 1-Week Post-implantation. Photomicrographs (optical microscopy) stained with hematoxylin/eosin of the membrane implantation area (dotted line) at ×40/×400 magnification. (**A**,**B**) Tea tree Experimental Group, (**C**,**D**) Furanone Experimental Group, (**E**,**F**) Pure PLGA Experimental Group, (**G**,**H**) Pratix Experimental Group.

After the 1-week histologic analysis (Figure 7), all membranes were clearly visible, remained physically structured and encapsulated within connective tissue.

All membrane groups exhibited a moderate inflammatory infiltrate, with exception of the tea tree-based PLGA group (Figure 7A,B) which showed an intense inflammatory infiltrate, predominantly mononuclear (especially with the recruitment of macrophages lymphocytes). A significant amount of multi-nucleated giant cells (see black arrows) was noticed on the experimental electrospun membranes surfaces (Figure 7A–F) contrasting with Pratix (Figure 7G,H). Fiber layer’s structure, originated from the manufacturing electrospinning process, were susceptible to accumulation of mesenchymal cells (Figure 7F). No fibrosis, fatty infiltrate, or tissue necrosis were observed in any experimental groups.

#### 3.3.2. 3-Weeks Post-Implantation

**Figure 8:**
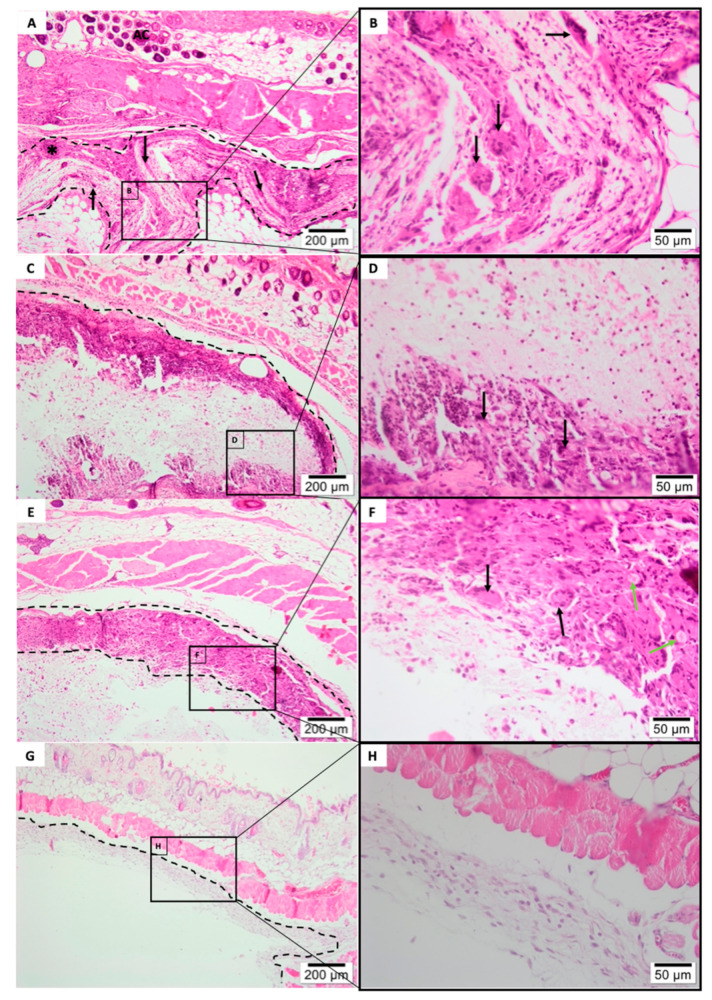
Histology of subcutaneous evaluation of 3-Weeks Post-implantation. Photomicrographs (optical microscopy) stained with hematoxylin/eosin of the membrane implantation area (dotted line) at ×40/×400 magnification. (**A**,**B**) Tea tree Experimental Group, (**C**,**D**) Furanone Experimental Group, (**E**,**F**) Pure PLGA Experimental Group, (**G**,**H**)Pratix Experimental Group.

After 3 weeks of subcutis implantation (Figure 8) electrospun membranes had reduced its structured stability, indicating a breakdown in the polymer chain, with furanone-based PLGA membrane (Figure 8 C,D) exhibiting the most stable rate of the degradability regarding nanofiber experimental groups. Pure PLGA membrane (Figure 8E,F) appeared fragmented with the presence of granulation tissue formation and multi-nucleated Giant cells within biomaterial phagocytized (see green arrows). Electrospun membranes (Figure 8A–F) were encapsulated within connective tissue and exhibited an intense inflammatory infiltrate, predominantly mononuclear with recruitment of macrophages and lymphocytes, with major multi-nucleated Giant cells colonization (black arrows). Inflammatory cells—mononuclear specifically, were also seen dispersed within the electrospun membranes.

The group related to the commercially available membrane (Figure 8G,H) showed a delicate connective tissue surrounding the biomaterial, containing scarce inflammatory infiltrate. Fibrosis, fatty infiltrate, and tissue necrosis remained absent.

#### 3.3.3. 9-Weeks Post-Implantation

**Figure 9:**
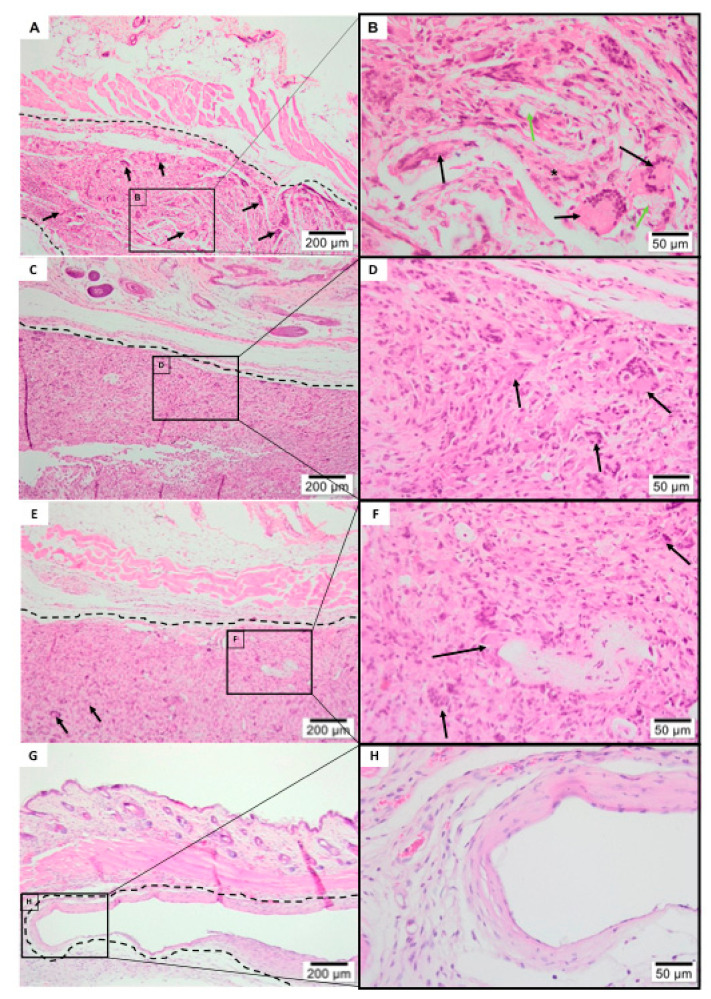
Histology of subcutaneous evaluation of 9-Weeks Post-implantation. Photomicrographs (optical microscopy) stained with hematoxylin/eosin of the membrane implantation area (dotted line) at ×40/×400 magnification. (**A**,**B**) Tea tree Experimental Group, (**C**,**D**) Furanone Experimental Group, (**E**,**F**) Pure PLGA Experimental Group, (**G**,**H**) Pratix Experimental Group.

At 9 weeks (Figure 9), all the PLGA electrospun membranes (Figure 9A–F) were almost completely degraded, presenting connective tissue on the implanted area alongside with granulation tissue formation. Residual traces of membrane material were also observed, frequently seen in the interior of the multi-nucleated Giant cells (Figure 9B; see green arrows). Pratix group membrane remained structured and encapsulated within connective tissue, presenting scarce inflammatory infiltrate in its surroundings (Figure 9G,H). No fibrosis, fatty infiltrate, or tissue necrosis were observed in any experimental period.

### 3.4. Semiquantitative Histological Analysis

**Figure 10:**
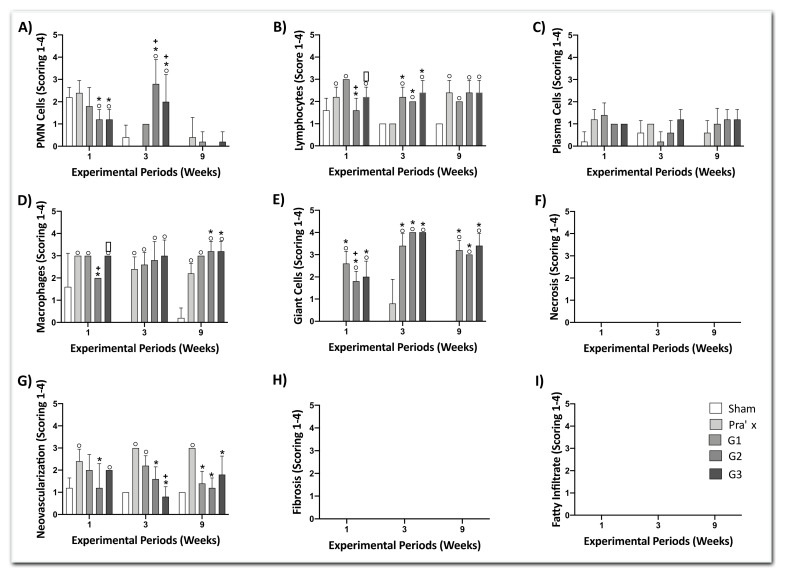
Inflammatory cells response (**A**–**E**) and overall tissue reaction (**F**–**I**) after experimental periods of 1, 3 and 9 weeks. Data ± standard deviation (*p* < 0.05). Sham group was considered an experimental control. (°) represents significantly difference when compared to the Sham group at same experimental period; (*) represents significantly difference when compared to the Pratix group at same experimental period; (+) represents significantly difference when compared to the G1 group at same experimental period; (*) represents significantly difference when compared to the G2 group at same experimental period.

Inflammatory cells response, composed of multi-nucleated Giant cells, macrophages, polymorphonuclear leukocytes, plasma cells, lymphocytes (shown in Figure 10A–E) and overall tissue response according to inflammatory parameters, such as neovascularization, fibrosis, and fatty infiltrate (Figure 10G–I) were evaluated and quantified. The degree of degeneration (debris) was also assessed by morphological alterations due to necrosis extension (Figure 10F).

Counting of the multi-nucleated giant cells (Figure 10E) and macrophages (Figure 10D) exhibited lavish numbers for electrospun fiber membrane (G1, G2 and G3) compared to Sham (no implantation) in all experimental periods. These cellular elements were characteristic of local inflammation due to the presence of a foreign body reaction.

In comparison with the other commercially available membrane (Pratix^®^), the major contrast was noted in the giant cells-count, in which Pratix presented no score, except for the third week experimental period. PLGA membrane Giant cells-counts range from 2 to 4, with no statistically significant difference between the three groups (neat PLGA and furanone and tea tree essential oil loaded PLGA fiber membranes).

Counting of polymorphonuclear leukocytes (PMNs) (Figure 10A) indicated less of these cells in contact with the electrospun membranes than with Pratix and Sham, in the first week. An increased in the PMNs was presented during the third week for G2 and G3, however all membranes have stabilized below score 1 for the last period.

Lymphocyte-count (Figure 10B) of all experimental groups reported statistically significant difference compared to Sham during the three experimental periods, except for G2 (1st week) and Pratix (3rd week). Furthermore, PLGA electrospun membranes showed significant difference between G1 and G2 and G2 and G3, exclusively in the first week.

Regarding neovascularization (Figure 10G), PLGA electrospun membranes exhibited significant lower scores compared to Pratix group during all the experimental periods. In addition, significantly difference between fiber membranes was noted for G1 and G3 throughout the third week.

Counts of plasm cells (Figure 10C) scored two less for all membranes during the three experimental periods.

Moreover, incidence of tissue necrosis (Figure 10F), fatty infiltrate (Figure 10I) and fibrosis (Figure 10H) have not been reported in this subcutis analysis in any of the experimental periods/groups.

Inflammatory reaction according to ISO 10993-6:2016 may be calculated by subtracting the group score of each period from the control (Sham) score leading to an estimated: Not irritating (0.0 to 2.9), slightly irritating (3.0 to 8.9), moderately irritating (9.0 to 15.0) or seriously irritating (>15). As stated, for the 1st week post implantation, G2, G3 and Pratix group presented a slightly irritant reaction, while G1exhibited a moderate irritant reaction. For the 3rd week post-implantation, the electrospun membranes G1, G2 and G3 showed a severe irritant reaction in contrast to moderate irritant reaction from Pratix group. Finally, for the 9th week after implantation, electrospun groups G1, G2 and G3 remained presenting a severe-irritant score. As well, Pratix group has continued exhibiting a moderate-irritant reaction.

## 4. Discussion

Biomaterials have been remarkably developed over the last years, targeting improvements in tissue engineering, immunotherapy, and drug delivery sphere [43,44,45,46]. Biological response, especially to implanted materials, are major concern to successfully achieve regenerative purposes. Experiments in this present study concentrated on antibiofilm activity and in vivo cellular reactions of resorbable polymer membranes fabricated by electrospinning process and incorporated with antibiofilm compounds for intended barrier tissue function.

A precursory study has reported on the electrospun fiber arrangement incorporated with tea tree essential oil and furanone, evaluating the morphology concerning the fiber diameters (36 μm) and results herein resemble past findings. Data obtained in this study indicated homogenous electrospun fibers and that the average fiber diameter slightly increased with addition of tea tree essential oil, from 0.61 ± 0.13 to 0.78 ± 0.14 µm, which could be explained to increase in solution viscosity [47]. Nevertheless, no significant effect on PLGA electrospun membranes morphology, compared to the addition groups, points towards reproducible production of fiber membranes [48]. The pore diameter of the electrospun fibers, furanone loaded PLGA membranes exhibited the highest measures for average of the pores (1.33 ± 0.59 µm) and maximum pore diameter of 2.97 µm. In conformity with previous reports, randomly oriented fibrous membranes that present pores size up to 5 µm has selective permeability preventing fibrous tissue infiltration and still allow oxygen, nutrients, and growth factors [13,49].

The overwhelming majority of absorbable membranes commercially available for tissue regeneration are based on polyesters or tissue-derived collagen. Since native ECM matrix is mainly composed of collagen, collagen-based membranes originated from human cadaver skin, amniotic placental membrane, and porcine or bovine tissues benefit from its exceptional biocompatibility and cell affinity [50]. The drawbacks based on collagen include insufficient mechanical properties and unpredictable degradation profile that may compromise the in vivo performance [51], furthermore, present risk of disease transmission [7]. Although different treatments have been reported to enhance biomechanical properties and stability of collagen matrices through natural crosslinking, the addition of plasma rich in growth factors and UV exposure [52,53,54], ethical and religious factors associated with high costs had directed to studies of alternative materials. Polymeric membranes, however, are biodegradable, biocompatible, and exhibit, in general, superior degradation rates. Yet, its biodegradation end-products may negatively affect cell response [9,55,56].

Medical device implantation in vivo of any biomaterial will elicit tissue and cellular response, that include inflammatory infiltration of numerous cells and it’s characterized by predominance of neutrophils in acute phase and monocytes in the chronic phase [57]. Macrophage and its fused morphologic derivation, the multinucleated giant cells, have shown to be early dominant responder and bind to almost all biomaterials implanted [58]. In this study, the results confirm this, as indicates a significant macrophage counts in all the materials implanted. Nevertheless, our findings exhibited that giant-cells were predominantly associated with electrospun fiber membranes, including the neat PLGA group, rather than the other commercially available membrane synthetized from common polymeric material, which culminated in a greater irritant-reaction score.

Santana et al. [5] reported a specific occurrence correlating topography and porosity of a double-sided polylactic acid membrane with degree of giant-cell response to the material, affirming that rough surface showed significantly more giant cells than the smooth surface of the same membrane. The idea of high surface to volume ratio and porous materials were already alluded to a superior rate of macrophages and giant-cells compared to smooth-surface implant materials [59]. In the light of this, we might assume that the topography and porosity of randomly nanofiber matrix, originated from electrospinning process, could potentially enhance giant-cell response. Moreover, additional previous studies regarding PLGA membranes have reported cellular inflammation and thin tissue fibrosis until complete resorption of the degradation products without jeopardize biomaterial safety [60].

Presence of macrophages in peri-implant tissue was universally understood as prejudicial to osseointegration [61]. Similarly, inflammatory cells and giant cells associated with barrier membranes were recognized as a compromised healing sign [62]. While the exact mechanism of macrophage polarization and interaction with biomaterials remains unclear, recent studies have suggested that macrophages might have no deleterious effect on final formation of osseointegration [63,64,65] and that inflammatory infiltration were not able to inhibit bone regeneration [5,60,66]. Since this present study was limited to the biologic response of the implanted membranes incorporated with antibiofilm, further work that addresses functionality in promoting bone regeneration will be conducted before extrapolating to more statements in that direction.

Nowadays, biofilms management is achieved by conventional systemic antibiotics, which alleviate the symptoms of infection by eliminating the planktonic microbial population yet is ineffective against those that remain within the biofilm [19], displaying an increase of up to a thousand times higher in resistance to antimicrobials [67]. Furanone and tea tree had biomaterial emerged as ideal antibacterial agents for preventing biofilm infection due to their broad spectrum, novel mechanisms of disrupting bacterial-communication system and consequently inhibiting biofilm formation in the early stages [68,69,70,71,72]. The antibiofilm activity of tea tree and furanone loaded PLGA electrospun fiber membranes were tested with *S*. *mutans*, and results confirmed preceding study [39] that indicated a significant biofilm inhibition on the electrospun membranes incorporated with compounds tested. Data analysis of biofilm by CFU counting detached from both tea tree and furanone loaded PLGA exhibited a significant reduction (*p* < 0.01) in the quantity of adhered viable cells compared to biofilm formed in the control (pure PLGA).

Few studies have reported on electrospun fiber systems loaded with tea tree oil, and only one for medical applications. Bai et al. [73] fabricated electrospun polycaprolactone mat coated with chitosan containing tea tree oil for functional wound dressings and investigated in animal mice model. Tea tree/chitosan group exhibited complete re-epithelization, with moderately suppression of neutrophils and greater blood vessels growing inward towards the injured area. Their findings resemble our results particularly in the 3rd week, which indicated a tendency for greater neovascularization in G1 compared with G3 (pure PLGA) and lower polymorphonuclear leukocytes count compared to all electrospun membrane groups. Based on this study, tea tree membranes showed a distinct irritation score from the other electrospun membranes only in 1st week, considered as moderately irritant while pure PLGA and furanone group presented a slightly irritant score.

Furanone as antibiofilm coating on polymeric biomaterials for medical devices have already been assessed. For instance, Baveja et al. [74] investigated the effect of a furanone compound on adhesion and slime production of *S. epidermidis* on biomaterials and its cytotoxicity on mammalian (murine) cells. Furanone surface coatings showed biofilm inhibition on all biomaterials studied and non-cytotoxic effect, after 48 h of exposure. This supports our previous findings indicating that furanones are not cytotoxic at levels that are antibacterial [39]. There is still insufficient data concerning safety in vivo*,* but our study suggested no difference in the overall irritation pattern between the furanone-based PLGA membrane and pure PLGA electrospun membranes (*p* < 0.05). In fact, counts of lymphocytes and macrophages demonstrated a regression tendency for furanone group, in comparison to neat PLGA and tea tree nanofibers, during the 1st week. In addition, polymorphonuclear leukocytes—including neutrophils activity, are minimal at 9th week. Our findings agree with Baveja et al. [75], who presented the idea that furanones themselves did not activate neutrophil integrin receptor expression or significant in vivo response in comparison to control, analyzing the biological performance of furanone compound covalently bound to polystyrene disks.

Biological response to a given material can show interdependence on several parameters and investigating in vivo particular aspects of the cell-surface interactions of new compounds can help to define key features for efficacy in future designing. Considering that there is a notable difference between animal and human clinical research and this should be kept in perspective, these novel electrospun fiber membranes incorporating antibiofilm compounds, furanone, and tea tree tested in this study, did not incite major inflammatory response compared to pure PLGA electrospun membranes, and no signs of necrosis, fibrosis, fatty tissue, or wound dehiscence were reported in any experimental group. Additional research is required associating antibacterial properties with bone regeneration response in vivo, with further ramifications to infected animal model, before inferring current findings to clinical practice.

## 5. Conclusions

In this study, novel PLGA membranes incorporating antibiofilm compounds were synthetized by the electrospinning process, to investigate antibiofilm, biological response and tissue cell integration. Incorporation of compounds presented in nanofibers exhibited significant biofilm inhibition and did not incite inflammatory response significantly different in comparison with pure PLGA electrospun membranes. The results obtained indicate promisor biological performance that combined with their antibiotic-free antibiofilm properties suggest that these membranes could be good candidates for biomedical implantable membranes in future clinical treatment of preventing biofilm-associated infections.

## References

[1] Dahlin C., Linde A., Gottlow J., Nyman S. (1988). Healing of Bone Defects by Guided Tissue Regeneration. Plast. Reconstr. Surg..

[2] Buser D., Brägger U., Lang N.P., Nyman S. (1990). Regeneration and enlargement of jaw bone using guided tissue regeneration. Clin. Oral Implant. Res..

[3] Takata T., Miyauchi M., Wang H.-L. (2001). Migration of osteoblastic cells on various guided bone regeneration membranes. Clin. Oral Implant. Res..

[4] Sculean A., Nikolidakis D., Schwarz F. (2008). Regeneration of periodontal tissues: Combinations of barrier membranes and grafting materials - biological foundation and preclinical evidence: A systematic review. J. Clin. Periodontol..

[5] de Santana R.B., de Mattos C.M.L., Francischone C.E., van Dyke T. (2010). Superficial Topography and Porosity of an Absorbable Barrier Membrane Impacts Soft Tissue Response in Guided Bone Regeneration. J. Periodontol..

[6] Rocchietta I., Fontana F., Simion M. (2008). Clinical outcomes of vertical bone augmentation to enable dental implant placement: A systematic review. J. Clin. Periodontol..

[7] Bottino M.C., Thomas V., Schmidt G., Vohra Y.K., Chu T.-M.G., Kowolik M.J., Janowski G. (2012). Recent advances in the development of GTR/GBR membranes for periodontal regeneration—A materials perspective. Dent. Mater..

[8] Gentile P., Chiono V., Carmagnola I., Hatton P.V. (2014). An Overview of Poly (lactic-co-glycolic) Acid (PLGA)-Based Biomaterials for Bone Tissue Engineering. Int. J. Mol. Sci..

[9] Takata T., Wang H.-L., Miyauchi M. (2001). Attachment, proliferation and differentiation of periodontal ligament cells on various guided tissue regeneration membranes. J. Periodontal Res..

[10] Passos P.C., Moro J., Barcelos R.C.S., Da Rosa H.Z., Vey L.T., Bürguer M.E., Maciel R.M., Danesi C.C., Edwards P.C., Bottino M.C. (2020). Nanofibrous antibiotic-eluting matrices: Biocompatibility studies in a rat model. J. Biomed. Mater. Res. Part B Appl. Biomater..

[11] Bottino M.C., Thomas V., Janowski G. (2011). A novel spatially designed and functionally graded electrospun membrane for periodontal regeneration. Acta Biomater..

[12] Unalan I., Endlein S.J., Slavik B., Buettner A., Goldmann W.H., Detsch R., Boccaccini A.R. (2019). Evaluation of Electrospun Poly (ε-Caprolactone)/Gelatin Nanofiber Mats Containing Clove Essential Oil for Antibacterial Wound Dressing. Pharmaceutics.

[13] Kim J.I., Hwang T.I., Aguilar L.E., Park C.H., Kim C.S. (2016). A Controlled Design of Aligned and Random Nanofibers for 3D Bi-functionalized Nerve Conduits Fabricated via a Novel Electrospinning Set-up. Sci. Rep..

[14] Li W.-J., Cooper J.A., Mauck R., Tuan R.S. (2006). Fabrication and characterization of six electrospun poly (α-hydroxy ester)-based fibrous scaffolds for tissue engineering applications. Acta Biomater..

[15] Cucchi A., Vignudelli E., Napolitano A., Marchetti C., Corinaldesi G. (2017). Evaluation of complication rates and vertical bone gain after guided bone regeneration with non-resorbable membranes versus titanium meshes and resorbable membranes. A randomized clinical trial. Clin. Implant. Dent. Relat. Res..

[16] Filippo Fontana D.D.S., Rocchietta I., Massimo Simion M.D. (2015). Complications in guided bone regeneration. Dental Implant Complications: Etiology, Prevention, and Treatment.

[17] Brady R.A., Leid J.G., Calhoun J.H., Costerton J.W., Shirtliff M.E. (2008). Osteomyelitis and the role of biofilms in chronic infection. FEMS Immunol. Med Microbiol..

[18] Marsh P., Lewis M., Williams D., Martin M. (2009). Oral Microbiology.

[19] Costerton J.W., Stewart P.S., Greenberg E.P. (1999). Bacterial Biofilms: A Common Cause of Persistent Infections. Science.

[20] Seebach E., Kubatzky K.F. (2019). Chronic Implant-Related Bone Infections—Can Immune Modulation be a Therapeutic Strategy?. Front. Immunol..

[21] Dai T., Vrahas M.S., Murray C.K., Hamblin M.R. (2012). Ultraviolet C irradiation: An alternative antimicrobial approach to localized infections?. Expert Rev. Anti-infective Ther..

[22] Scaffaro R., Maio A., D’Arrigo M., Lopresti F., Marino A., Bruno M., Nostro A. (2020). Flexible mats as promising antimicrobial systems via integration of *Thymus capitatus* (L.) essential oil into PLA. Futur. Microbiol..

[23] Scaffaro R., Gulino F.E., Lopresti F. (2020). Structure–property relationship and controlled drug release from multiphasic electrospun carvacrol-embedded polylactic acid/polyethylene glycol and polylactic acid/polyethylene oxide nanofiber mats. J. Ind. Text..

[24] Eleung V., Edufour D., Lévesque C.M. (2015). Death and survival in Streptococcus mutans: Differing outcomes of a quorum-sensing signaling peptide. Front. Microbiol..

[25] Xavier J., Geremias T., Montero J., Vahey B., Benfatti C., Souza J., Magini R., Pimenta A. (2016). Lactam inhibiting Streptococcus mutans growth on titanium. Mater. Sci. Eng. C.

[26] Montero J.F., Tajiri H.A., Barra G.M.D.O., Fredel M.C., Benfatti C.A., Magini R.S., Pimenta A.L., Souza J.C. (2017). Biofilm behavior on sulfonated poly(ether-ether-ketone) (sPEEK). Mater. Sci. Eng. C.

[27] Miller M.B., Bassler B. (2001). Quorum Sensing in Bacteria. Annu. Rev. Microbiol..

[28] Jang Y.-J., Choi Y.-J., Lee S.-H., Jun H.-K., Choi B.-K. (2013). Autoinducer 2 of Fusobacterium nucleatum as a target molecule to inhibit biofilm formation of periodontopathogens. Arch. Oral Biol..

[29] Bjarnsholt T., Givskov M. (2007). Quorum-sensing blockade as a strategy for enhancing host defences against bacterial pathogens. Philos. Trans. R. Soc. B Biol. Sci..

[30] Cadavid E., Echeverri F. (2019). The Search for Natural Inhibitors of Biofilm Formation and the Activity of the Autoinductor C6-AHL in Klebsiella pneumoniae ATCC 13884. Biomolecules.

[31] Kayumov A., Khakimullina E.N., Sharafutdinov I., Trizna E.Y., Latypova L.Z., Lien H.T., Margulis A.B., Bogachev M., Kurbangalieva A.R. (2014). Inhibition of biofilm formation in Bacillus subtilis by new halogenated furanones. J. Antibiot..

[32] Kim C., Kim J., Park H.Y., Park H.J., Lee J.H., Kim C.K., Yoon J. (2008). Furanone derivatives as quorum-sensing antagonists of Pseudomonas aeruginosa. Appl. Microbiol. Biotechnol..

[33] Park J.S., Ryu E.-J., Li L., Choi B.-K., Kim B.M. (2017). New bicyclic brominated furanones as potent autoinducer-2 quorum-sensing inhibitors against bacterial biofilm formation. Eur. J. Med. Chem..

[34] He Z., Wang Q., Hu Y., Liang J., Jiang Y., Ma R., Tang Z., Huang Z. (2012). Use of the quorum sensing inhibitor furanone C-30 to interfere with biofilm formation by Streptococcus mutans and its luxS mutant strain. Int. J. Antimicrob. Agents.

[35] Bordini E.A.F., Tonon C.C., Francisconi R.S., Magalhães F.A.C., Huacho P.M.M., Bedran T.L., Pratavieira S., Spolidorio L.C., Spolidorio D.P. (2018). Antimicrobial effects of terpinen-4-ol against oral pathogens and its capacity for the modulation of gene expression. Biofouling.

[36] Comin V.M., Lopes L.Q., Quatrin P.M., de Souza M.E., Bonez P.C., Pintos F.G., Raffin R., Vaucher R.D.A., Martinez D.S.T., Santos R.C.V. (2016). Influence of Melaleuca alternifolia oil nanoparticles on aspects of Pseudomonas aeruginosa biofilm. Microb. Pathog..

[37] de Souza M.E., Clerici D.J., Verdi C.M., Fleck G., Quatrin P.M., Spat L.E., Bonez P.C., Dos Santos C.F., Antoniazzi R.P., Zanatta F.B. (2017). Antimicrobial activity of Melaleuca alternifolia nanoparticles in polymicrobial biofilm in situ. Microb. Pathog..

[38] Kolenbrander P.E. (2000). Oral Microbial Communities: Biofilms, Interactions, and Genetic Systems. Annu. Rev. Microbiol..

[39] Geremias T.C., Batistella M.A., Magini R.R.S., de Souza S.M.A.G.U., Franco C.V., Barbosa L.C.A., Pereira U.A., Hinestroza J.P., Pimenta A.L., de Souza A.A.U. (2021). Functionalization of poly (lactic-co-glycolic acid) nanofibrous membranes with antibiofilm compounds. Can. J. Chem. Eng..

[40] Kilkenny C., Browne W.J., Cuthill I.C., Emerson M., Altman D.G. (2010). Improving bioscience research reporting: The ARRIVE guidelines for reporting animal research. J. Pharmacol. Pharmacother..

[41] Smith A.J., Clutton R.E., Lilley E., Hansen K.E.A., Brattelid T. (2017). PREPARE: Guidelines for planning animal research and testing. Lab. Anim..

[42] Sealed Envelope. https://www.stat.ubc.ca/~rollin/stats/ssize/n2.html.

[43] Banerjee S., Bagchi B., Pal K., Bhandary S., Kool A., Hoque N.A., Biswas P., Thakur P., Das K., Karmakar P. (2020). Essential oil impregnated luminescent hydroxyapatite: Antibacterial and cytotoxicity studies. Mater. Sci. Eng. C.

[44] Cheng Y., Mei S., Kong X., Liu X., Gao B., Chen B., Wu J. (2021). Long-term antibacterial activity of a composite coating on titanium for dental implant application. J. Biomater. Appl..

[45] Yüksel E., Karakeçili A., Demirtaş T.T., Gümüşderelioğlu M. (2016). Preparation of bioactive and antimicrobial PLGA membranes by magainin II/EGF functionalization. Int. J. Biol. Macromol..

[46] Peppas N.A., Narasimhan B. (2014). Mathematical models in drug delivery: How modeling has shaped the way we design new drug delivery systems. J. Control. Release.

[47] Mori C.L.S.D.O., Dos Passos N.A., Oliveira J., Altoé T.F., Mori F.A., Mattoso L.H.C., Scolforo J.R., Tonoli G. (2015). Nanostructured Polylactic Acid/Candeia Essential Oil Mats Obtained by Electrospinning. J. Nanomater..

[48] Unalan I., Slavik B., Buettner A., Goldmann W.H., Frank G., Boccaccini A.R. (2019). Physical and Antibacterial Properties of Peppermint Essential Oil Loaded Poly (ε-caprolactone) (PCL) Electrospun Fiber Mats for Wound Healing. Front. Bioeng. Biotechnol..

[49] Oh S.H., Kim J.H., Song K.S., Jeon B.H., Yoon J.H., Seo T.B., Namgung U., Lee I.W., Lee J.H. (2008). Peripheral nerve regeneration within an asymmetrically porous PLGA/Pluronic F127 nerve guide conduit. Biomaterials.

[50] Behring J., Junker R., Walboomers X.F., Chessnut B., Jansen J.A. (2008). Toward guided tissue and bone regeneration: Morphology, attachment, proliferation, and migration of cells cultured on collagen barrier membranes. A systematic review. Odontology.

[51] Park J.K., Yeom J., Oh E.J., Reddy M., Kim J.Y., Cho D.-W., Lim H.P., Kim N.S., Park S.W., Shin H.-I. (2009). Guided bone regeneration by poly (lactic-co-glycolic acid) grafted hyaluronic acid bi-layer films for periodontal barrier applications. Acta Biomater..

[52] Bottino M.C., Thomas V., Jose M.V., Dean D.R., Janowski G.M. (2010). Acellular dermal matrix graft: Synergistic effect of rehydration and natural crosslinking on mechanical properties. J. Biomed. Mater. Res. Part B Appl. Biomater..

[53] Ratiu C., Brocks M., Costea T., Moldovan L., Cavalu S. (2019). PRGF-Modified Collagen Membranes for Guided Bone Regeneration: Spectroscopic, Microscopic and Nano-Mechanical Investigations. Appl. Sci..

[54] Cavalu S., Roiu G., Pop O., Heredea D., Costea T., Costea C. (2021). Nano-Scale Modifications of Amniotic Membrane Induced by UV and Antibiotic Treatment: Histological, AFM and FTIR Spectroscopy Evidence. Materials.

[55] Kikuchi M., Koyama Y., Yamada T., Imamura Y., Okada T., Shirahama N., Akita K., Takakuda K., Tanaka J. (2004). Development of guided bone regeneration membrane composed of β-tricalcium phosphate and poly (l-lactide-co-glycolide-co-ε-caprolactone) composites. Biomaterials.

[56] Tessmar J.K.V., Holland T.A., Mikos A.G., Ma P., Elisseeff J. (2006). Salt Leaching for Polymer Scaffolds: Laboratory-Scale Manufacture of Cell Carriers. Scaffolding in Tissue Engineering.

[57] Sheikh Z., Brooks P.J., Barzilay O., Fine N., Glogauer M. (2015). Macrophages, Foreign Body Giant Cells and Their Response to Implantable Biomaterials. Materials.

[58] Xia Z., Triffitt J.T. (2006). A review on macrophage responses to biomaterials. Biomed. Mater..

[59] Mitragotri S., Lahann J. (2009). Physical approaches to biomaterial design. Nat. Mater..

[60] Hoornaert A., D’Arros C., Heymann M.-F., Layrolle P. (2016). Biocompatibility, resorption and biofunctionality of a new synthetic biodegradable membrane for guided bone regeneration. Biomed. Mater..

[61] Rich A., Harris A. (1981). Anomalous preferences of cultured macrophages for hydrophobic and roughened substrata. J. Cell Sci..

[62] Rothamel D., Schwarz F., Sculean A., Herten M., Scherbaum W., Becker J. (2004). Biocompatibility of various collagen membranes in cultures of human PDL fibroblasts and human osteoblast-like cells. Clin. Oral Implant. Res..

[63] Miron R.J., Bosshardt D.D. (2016). OsteoMacs: Key players around bone biomaterials. Biomaterials.

[64] Wang X., Li Y., Feng Y., Cheng H., Li D. (2020). The role of macrophages in osseointegration of dental implants: An experimental study in vivo. J. Biomed. Mater. Res. Part A.

[65] Miron R.J., Zohdi H., Fujioka-Kobayashi M., Bosshardt D.D. (2016). Giant cells around bone biomaterials: Osteoclasts or multi-nucleated giant cells?. Acta Biomater..

[66] Böstman O.M., Pihlajamäki H. (2000). Adverse tissue reactions to bioabsorbable fixation devices. Clin. Orthop. Relat. Res..

[67] Ceri H., Olson M., Stremick C., Read R.R., Morck D., Buret A. (1999). The Calgary Biofilm Device: New Technology for Rapid Determination of Antibiotic Susceptibilities of Bacterial Biofilms. J. Clin. Microbiol..

[68] Sharafutdinov I.S., Pavlova A.S., Akhatova F.S., Khabibrakhmanova A.M., Rozhina E.V., Romanova Y.J., Fakhrullin R., Lodochnikova O.A., Kurbangalieva A.R., Bogachev M.I. (2019). Unraveling the Molecular Mechanism of Selective Antimicrobial Activity of 2 (5H)-Furanone Derivative against Staphylococcus aureus. Int. J. Mol. Sci..

[69] Proctor C.R., McCarron P.A., Ternan N.G. (2020). Furanone quorum-sensing inhibitors with potential as novel therapeutics against Pseudomonas aeruginosa. J. Med Microbiol..

[70] Brun P., Bernabè G., Filippini R., Piovan A. (2019). In Vitro Antimicrobial Activities of Commercially Available Tea Tree (Melaleuca alternifolia) Essential Oils. Curr. Microbiol..

[71] Song Y.-M., Zhou H.-Y., Wu Y., Wang J., Liu Q., Mei Y.-F. (2020). In Vitro Evaluation of the Antibacterial Properties of Tea Tree Oil on Planktonic and Biofilm-Forming Streptococcus mutans. AAPS PharmSciTech.

[72] Casarin M., Pazinatto J., Santos R.C.V., Zanatta F.B. (2018). Melaleuca alternifolia and its application against dental plaque and periodontal diseases: A systematic review. Phytother. Res..

[73] Bai M.Y., Chou T.C., Tsai J.C., Yu W.C. (2014). The effect of active ingredient-containing chitosan/polycaprolactone nonwoven mat on wound healing: In vitro and in vivo studies. J. Biomed. Mater. Res. Part A.

[74] Baveja J., Willcox M., Hume E., Kumar N., Odell R., Poole-Warren L. (2004). Furanones as potential anti-bacterial coatings on biomaterials. Biomaterials.

[75] Baveja J., Li G., Nordon R., Hume E., Kumar N., Willcox M., Poole-Warren L. (2004). Biological performance of a novel synthetic furanone-based antimicrobial. Biomaterials.

